# The association between family health and frailty in preoperative patients with gastric cancers: the mediating role of health literacy and physical activity

**DOI:** 10.3389/fpubh.2025.1541688

**Published:** 2025-05-23

**Authors:** Hanjia Xin, Chaozhu He, Yingying Gu, Xiuxiu Ma, Ziying Xiang, Jingjing Gong

**Affiliations:** ^1^School of Nursing, Jiangxi Medical College, Nanchang University, Nanchang, China; ^2^Department of General Surgery, The First Affiliated Hospital of Nanchang University, Nanchang, China

**Keywords:** gastric cancer, preoperative care, frailty, family health, health literacy, physical activity, mediating role

## Abstract

**Introduction:**

Frailty is prevalent among preoperative gastric cancer (GC) patients and significantly affects surgical risk and long-term recovery. Family health may hold substantial potential for mitigating frailty, although the mechanisms underlying this effect remain unclear. This study aims to investigate the impact of family health on frailty in preoperative GC patients, and the mediating effects of health literacy and physical activity.

**Methods:**

A total of 240 patients scheduled for radical gastrectomy at a tertiary hospital in China were surveyed using Family Health Scale (FHS), Health Literacy Scale (HLS-SF), International Physical Activity Questionnaire (IPAQ-7), and Tilburg Frailty Indicator (TFI). Data were analyzed using independent *t*-tests, *χ*^2^ tests, Pearson’s correlation, and binary logistic regression. Mediation analysis with Structural Equation Modeling (SEM) was then applied to explore the relationships between variables.

**Results:**

Family health in preoperative GC patients was negatively correlated with frailty (*r* = −0.791, *p* < 0.01) and positively correlated with both health literacy (*r* = 0.806, *p* < 0.01) and physical activity (*r* = 0.464, *p* < 0.01). Mediating effect analysis indicated that the direct effect of family health on frailty was −0.837, while health literacy and physical activity served as partial mediators in this relationship, with indirect effects of −0.332 and −0.095 (both *p* < 0.01), respectively. The mediating effects accounted for 33.83% of the total effect.

**Conclusion:**

Family health directly affects frailty and also exerts an indirect impact through the mediators of health literacy and physical activity. These findings suggest that healthcare professionals should focus on vulnerable populations with low family health and implement family-centered preoperative frailty interventions. Guiding GC patients to improve health literacy and engage in personalized family-based exercises can help delay or reverse preoperative frailty, promoting long-term recovery outcomes.

## 1 Introduction

According to the latest data released by the International Agency for Research on Cancer (IARC), there are approximately 968,000 new cases of gastric cancer (GC) and 660,000 deaths globally (1). In China, GC ranks third in both incidence and mortality among all cancers, with an overall 5-year survival rate of less than 50%, making it a significant public health concern (2). Currently, surgical resection remains the preferred approach for achieving radical cure and improving long-term survival in GC patients. As the population ages at an unprecedented rate, individuals aged 60 and older now account for 70.8% of all GC cases, with an increasing proportion of patients in this age group undergoing gastrectomy. However, due to a combination of factors such as aging, tumor-related metabolic disturbances, and nutritional and skeletal muscle abnormalities, preoperative GC patients often exhibit frailty symptoms, including weight loss, fatigue, reduced grip strength and decreased pace (3). Frailty refers to a homeostatic imbalance of dysfunction, decreased physiological reserve, increased vulnerability, and decreased anti-stress capacity after a stressor event (4), and is clinically dynamic and reversible (5). A systematic review (6) reported that the incidence of frailty in GC patients ranges from 10 to 71%, with an overall prevalence of 29%. Preoperative frailty not only increases surgical risks (7), but also predisposes patients to a range of adverse health outcomes postoperatively, including increased complications, prolonged hospital stays, disability, and mortality, which significantly affect long-term recovery (8, 9). Therefore, preoperative frailty management in GC patients is crucial.

In recent years, the concept of “family health” has garnered increasing attention within the academic community, with a growing number of studies both domestically and internationally focusing on family-based health promotion and disease management (10, 11). “Family health” refers to a resource at the family unit level, arising from the interplay of the health, abilities, behaviors, personalities, and interactions of each family member, as well as the family’s physical, social, emotional, economic, and medical resources (12). Numerous studies have underscored the indispensable role of families in providing care for individuals with chronic diseases, disabilities, and frailty. In fact, the economic value generated by family-based caregiving is estimated to be 2–6 times greater than that of formal healthcare systems (13). Despite this substantial contribution, the potential of family-centered approaches to mitigate frailty, optimize public health resource allocation, and curtail healthcare costs remains largely untapped. Currently, domestic frailty management strategies predominantly concentrate on individual-level interventions, thereby overlooking the family as a central health-promoting entity.

Health literacy and physical activity, as modifiable cognitive and behavioral factors, have demonstrated efficacy in improving individual frailty outcomes, offering a crucial avenue for understanding the relationship between family health and frailty. Health literacy is defined as the ability of individuals to access, comprehend, and utilize basic health information to promote their own health (14). This capability is often influenced by factors such as family structure, income level, and the educational background of family members (15). Research indicates that low health literacy is associated with patients’ inadequate understanding of their disease status and poor self-care abilities, which can adversely affect disease management and health outcomes in individuals with chronic conditions (16). Therefore, enhancing patients’ health literacy is anticipated to help prevent the onset of preoperative frailty. Furthermore, physical activity is a key and significant indicator of bodily functions. Research has demonstrated that supportive interactions and shared values regarding healthy behaviors within a family can influence the level of physical activity individuals engage in Wunsch et al. (17). Previous studies have also established that exercise frequency serves as a crucial predictor of frailty (18). However, previous research has yet to explore the mediating effects of these factors in the context of how family health influences preoperative frailty.

In this study, we hypothesized that family health is negatively associated with frailty, and that health literacy and physical activity partially mediate the relationship between the two. To test this hypothesis, we conducted a cross-sectional survey at a tertiary hospital in China. The study aimed to provide clinicians with a cost-effective and evidence-based strategy to reduce preoperative frailty risk in GC patients while offering theoretical insights for the development of family-friendly healthcare policies and support systems, particularly in the face of an aging population and increasingly strained healthcare resources.

## 2 Materials and methods

### 2.1 Study design and participants

This cross-sectional study was conducted in accordance with STROBE Statement (19). A convenience sampling method was employed to select 240 patients scheduled for radical gastrectomy at the First Affiliated Hospital of Nanchang University in China, between November 2023 and April 2024. Inclusion criteria include: Aged ≥ 18 years; A confirmed diagnosis of primary GC according to the China Standardization for Diagnosis and Treatment of Gastric Cancer (2022 edition) (20); United States Society of anesthesiologists (ASA) classification (21) Class I–III; Waiting for the first radical gastrectomy at our hospital. Exclusion criteria included: preoperative chemoradiotherapy; Concomitant other primary malignancies or serious medical diseases; critically ill and in the acute phase; Have a history of psychiatric illness or cognitive dysfunction; Severe limitations in hearing, vision, or speech impairment. The sample size was determined based on the empirical criterion that the sample size for Structural Equation Modeling (SEM) should be ≥150 (22). All study subjects gave informed consent and voluntarily participated in this study.

### 2.2 Survey instruments

#### 2.2.1 General information questionnaire

The General Information Questionnaire was designed to collect general demographic and health-related data of patients. Demographic data include gender, age, marital status, number of offspring, place of residence, highest educational level, household per capita monthly income, etc.; Health-related information included Body Mass Index (BMI), tumor TNM stage, number of comorbidity and number of medicines.

#### 2.2.2 Family health scale (FHS)

The FHS was developed by Crandall et al. (23) and revised into Chinese by Wang et al. (24), to assess participants’ family health functioning. The scale includes 4 dimensions of family social and emotional wellbeing process, social approach to family health, family health resources, and social support outside the family, with a total of 10 items, and is scored on a 5-level Likert scale (1 = strongly disagree, 2 = somewhat disagree, 3 = neither agree nor disagree, 4 = somewhat agree, 5 = strongly agree), with questions 6, 9, and 10 being scored backwards. The total score of the scale is between 10 and 50 points, and the higher the score, the better the level of family health. The Cronbach’s *α* coefficient was 0.830 for the Chinese version of the scale. This study Cronbach’s *α* coefficient of 0.814.

#### 2.2.3 Health literacy scale short form (HLS-SF4)

Originally developed by Duong et al. (15) and subsequently adapted and simplified by domestic scholars Sun et al. (25). It was simplified to a 4-item version using Classical Test Theory and the Mokken model to measure public health literacy. The HLS-SF4 comprises three dimensions: healthcare, health promotion, and disease prevention. It utilizes a 4-point Likert scale for scoring (1 = very difficult, 2 = difficult, 3 = easy, 4 = very easy), with total scores ranging from 4 to 16, where a higher scores indicate a greater level of health literacy. The HLS-SF4 has demonstrated strong reliability and validity, with a Cronbach’s *α* coefficient of 0.842 and an intraclass correlation coefficient (ICC) for criterion validity of 0.892 (95% CI: 0.886–0.899), making it a reliable and concise tool for measuring health literacy. In our research, the Cronbach’s *α* coefficient for the HLS-SF4 was 0.801.

#### 2.2.4 The international physical activity questionnaire (IPAQ-7)

The IPAQ-7 (26) assesses participants’ activities in terms of heavy physical activity, moderate physical activity, and walking in the past week. It contains a total of 7 items. The exercise duration is calculated by summing all the activity time (minutes). Physical activity levels were assessed based on previous studies, with 150 min of exercise per week deemed to be a healthy benchmark (27). If individuals engaged in physical activity for less than 150 min per week, their activity levels were not considered sufficient.

#### 2.2.5 Tilburg frailty indicator (TFI)

The TFI developed by Netherlands Tiburg University scholar Gobbens et al. (28) based on the frailty integration model, including 3 dimensions of physical frailty, psychological frailty and social frailty, a total of 15 self-report items, 11 items in the scale are “yes, no” dichotomous options, which are scored 0 or 1 respectively, and the remaining 4 items are “yes, sometimes, no” tricategorical options, with intermediate values 0.5 points. The total score of the scale is the sum of the scores of each item, with a range of 0–15 points, and ≥ 5 points are frailty, and the higher the score, the more severe the frailty. The TFI scale has been validated by domestic scholars, showing good reliability and validity (29). In this study, the Cronbach’s *α* coefficient was 0.830.

### 2.3 Survey methodology

A one-on-one, face-to-face questionnaire survey was conducted with GC patients 24 h before surgery by systematically trained surveyors. Prior to the survey, the purpose and methodology of completing the questionnaires were clearly explained to the patients using standardized guidance, and data collection commenced only after obtaining informed consent. For participants who were physically weak or had low educational levels and were unable to complete the questionnaires independently, investigators read the questions aloud one by one and recorded the responses on their behalf. After completing the questionnaire, participants were informed of their responses to verify accuracy, and any discrepancies were addressed by repeating the questions and answers as necessary. A total of 240 questionnaires were distributed, and 223 valid responses were collected, yielding an effective response rate of 92.92%.

### 2.4 Statistical methods

Data were entered into Excel using a two-person double-entry verification method. SPSS 26.0 and Amos 23.0 software were employed for data organization and statistical analysis. Measurement data conforming to a normal distribution were expressed as Mean ± Standard Deviation (SD). The independent samples *t*-test was employed for group comparisons. Enumeration data were presented as frequencies and percentages, with the *χ*^2^ test applied for analysis. Point-biserial correlation was used to analyze the relationship between the binary variable and continuous variables, while Pearson’s correlation coefficient was employed for the relationships among the continuous variables. Variables that demonstrated statistically significant differences in univariate analysis were included in a binary logistic regression analysis, with a significance level set at *α* = 0.05. In the analysis of mediating effects using SEM, frailty was treated as the dependent variable, family health as the independent variable, and health literacy and physical activity as mediating variables. The bootstrap method was utilized for significance testing; a 95% confidence interval that did not include 0 indicated the establishment of a partial mediation effect.

## 3 Results

### 3.1 General demographic data of preoperative GC patients and single factor analysis of frailty

A total of 223 patients were included in this study, of which the age of the survey group was (64.41 ± 9.75) years, mainly older adult patients. There were 147 males (65.9%) and 76 females (34.1%). As shown in Table 1, frailty scores varied by age, gender, highest educational level, household per capita monthly income, tumor TNM stage, number of comorbidity, number of medicines, and physical activity (*p* < 0.05).

**Table 1:** General demographic data of preoperative GC patients and single factor analysis of frailty (*n* = 223).

Variables	All (*N* = 223)	Non-frail (*n* = 120)	Frail (*n* = 103)	*χ*^2^/*t*	*p*
Age (years), Mean ± SD	64.41 ± 9.75	59.93 ± 8.69	69.63 ± 8.23	8.515	<0.001*
Gender, *n* (%)				5.008	0.025*
Male	147 (65.9)	87 (72.5)	60 (58.3)		
Female	76 (34.1)	33 (27.5)	43 (41.7)		
Marital status, *n* (%)				1.162	0.281
Married	177 (79.4)	92 (76.7)	85 (82.5)		
Divorced/widowed/unmarried	46 (20.6)	28 (23.3)	18 (17.5)		
Number of offspring, *n* (%)				1.933	0.380
0	12 (5.4)	6 (5.0)	6 (5.8)		
1	34 (15.2)	22 (18.3)	12 (11.7)		
≥2	177 (79.4)	92 (76.7)	85 (82.5)		
Place of residence, *n* (%)				0.124	0.724
Urban	86 (38.6)	45 (37.5)	41 (39.8)		
Rural	137 (61.4)	75 (62.5)	62 (60.2)		
Highest educational level, *n* (%)				38.875	<0.001*
No formal education	31 (13.9)	7 (5.8)	24 (23.3)		
Junior high school	92 (41.3)	37 (30.8)	55 (53.4)		
High School	77 (34.5)	58 (48.3)	19 (18.4)		
Bachelor’s degree or above	23 (10.3)	18 (15.0)	5 (4.9)		
Household per capita monthly income (yuan), *n* (%)				7.755	0.021*
≤3,000	68 (30.5)	30 (25.0)	38 (36.9)		
3,001–6,000	124 (55.6)	77 (64.2)	47 (45.6)		
≥6,001	31 (13.9)	13 (10.8)	18 (17.5)		
BMI (kg/m^2^), Mean ± SD	22.34 ± 3.08	22.69 ± 2.71	21.94 ± 3.43	1.801	0.073
Tumor TNM stage, *n* (%)				43.348	<0.001*
I	35 (15.7)	31 (25.8)	4 (3.9)		
II	92 (41.3)	60 (50.0)	32 (31.1)		
≥III	96 (43.0)	29 (24.2)	67 (65.0)		
Number of comorbidity ≥ 2, *n* (%)				41.366	<0.001*
No	139 (62.3)	98 (81.7)	41 (39.8)		
Yes	84 (37.7)	22 (18.3)	62 (60.2)		
Number of medicines, *n* (%)				35.596	<0.001*
0	94 (42.2)	67 (55.8)	27 (26.2)		
1–2	78 (35.0)	43 (35.8)	35 (34.0)		
≥3	51 (22.9)	10 (8.3)	41 (39.8)		
Physical activity (minutes per week), *n* (%)				62.967	<0.001*
<150	87 (39.0)	18 (15.0)	69 (67.0)		
≥150	136 (61.0)	102 (85.0)	34 (33.0)		

*p < 0.05.

### 3.2 Family health, health literacy and frailty scale and its dimension scores of preoperative GC patients

The scores of the 3 scales and their dimensions are shown in Table 2. Among 223 preoperative GC patients, 103 (46.2%) were frail and 120 (53.8%) were non-frail.

**Table 2:** Family health, health literacy and frailty scale and its dimension scores of preoperative GC patients (*n* = 223).

Variables	Term	Mean ± SD
Family health	Family health scale	35.83 ± 4.98
	Family, social, or emotional health processes	12.43 ± 1.67
	Family healthy lifestyle	7.99 ± 1.24
	Family health resources	9.90 ± 2.49
	Family external social support	5.52 ± 2.51
Health literacy	Health Literacy Scale Short Form	8.12 ± 2.36
	Health care	3.59 ± 1.48
	Health promotion	2.64 ± 1.04
	Disease prevention	1.89 ± 0.91
Frailty	Tilburg frailty indicator	5.09 ± 2.46
	Physical frailty	2.76 ± 1.56
	Psychological frailty	1.75 ± 0.94
	Social frailty	0.58 ± 0.61

SD, standard deviation.

### 3.3 Correlation analysis of family health, health literacy, physical activity and frailty in preoperative GC patients

Correlation analysis showed that family health was negatively correlated with frailty (*r* = −0.791, *p* < 0.01) and positively correlated with both health literacy (*r* = 0.806, *p* < 0.01) and physical activity (*r* = 0.464, *p* < 0.01), as shown in Table 3.

**Table 3:** Correlation analysis of family health, health literacy, physical activity and frailty in preoperative GC patients.

Variables	Family health	Health literacy	Physical activity	Frailty
Family health	1			
Health literacy	0.806^a^**	1		
Physical activity	0.464^b^**	0.515^b^**	1	
Frailty	−0.791^a^**	−0.828^a^**	−0.564^b^**	1

**p < 0.01, a = Pearson correlation coefficient, b = point-biserial correlation coefficient.

### 3.4 Multivariate regression analysis of frailty in preoperative GC patients

Multivariate regression analysis used the frailty score as the dependent variable, and the variables with statistically significant differences in the univariate analysis included the independent variables. The specific variable assignments are listed in Table 4. The results of the analysis showed that physical activity, health literacy, and family health were the influencing factors for frailty in preoperative GC patients (*p* < 0.01; Table 5).

**Table 4:** Independent variable assignment.

Variable	Assignment method
Gender	Male = 1, Female = 2
Age (years)	Original value input
Marital status	Married = 1
	Divorced/widowed/unmarried = 2
Number of offspring	0 = 0, 1 = 1, ≥2 = 2
Place of residence	Urban = 1, Rural = 2
Highest educational level	No formal education = 1, Junior high school = 2, High School = 3, Bachelor’s degree or above = 4
Household per capita monthly income (yuan)	≤3,000 = 1, 3,001–6,000 = 2, 6,001 = 3
BMI (kg/m^2^)	Original value input
Tumor TNM stage	I = 1, II = 2, ≥III = 3
Number of comorbidity ≥ 2	No = 0, Yes = 1
Number of medicines	0 = 0, 1–2 = 1, ≥3 = 2
Physical activity (minutes per week)	<150 = 1, ≥150 = 2

**Table 5:** Multivariate regression analysis of frailty in preoperative GC patients.

Variable	*B* value	Standard error	Wald *χ*^2^	*p* value	*OR* value	Bootstrap 95%CI	
						Lower	Upper
Constant quantity	28.250	8.881	10.118	0.001			
Physical activity	−2.380	0.954	6.230	0.013	0.093	0.014	0.600
Health literacy	−1.405	0.498	7.949	0.005	0.245	0.092	0.652
Family health	−0.451	0.168	7.208	0.007	0.637	0.458	0.885

### 3.5 Mediating effects of family health, health literacy and physical activity on frailty in preoperative GC patients

**Figure 1:**
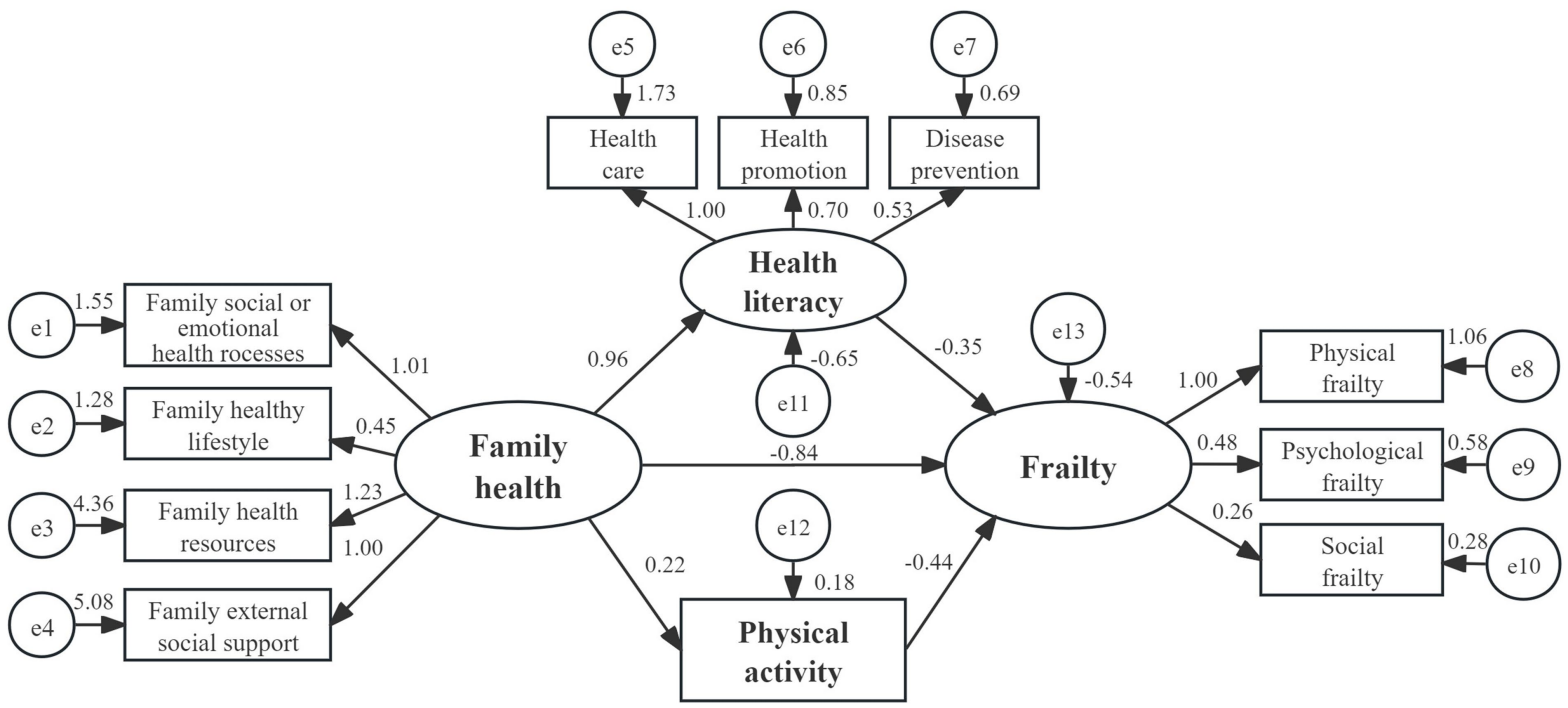
Mediating analysis of family health, health literacy, physical activity, and frailty.

A structural equation model was constructed using Amos 23.0 software. Family health was taken as independent variable, health literacy and physical activity as intermediate variable, and frailty as dependent variable. Maximum likelihood method was used to fit the hypothetical model. The results showed that the model fit well: *χ*^2^/df = 3.121, RMSEA = 0.049, IFI = 0.919, TLI = 0.873, CFI = 0.918. The confidence interval for the mediating effect was then calculated using the bias corrected Bootstrap method, limiting 5,000 repeated samples to construct a 95% bias corrected confidence interval. The results showed that the indirect effects of family health through health literacy (95% CI = −0.597 to −0.032), physical activity (95% CI = −0.232 to −0.033) and frailty were significant, the mediating effect value is −0.332 and −0.095 (both *p* < 0.01), accounting for 33.83% of the total effect; meanwhile, the direct effect of family health to frailty is also significant (95% CI = −1.401 to −0.549), the effect value is −0.837 (*p* < 0.01), accounting for 66.17% of the total effect (Figure 1 and Table 6).

**Table 6:** Mediating effects of family health, health literacy and physical activity on frailty in preoperative GC patients.

Path	Effect	Effect size (%)	SE	*p* value	Bootstrap 95%CI	
					Lower	Upper
Indirect effects	−0.428	33.83	0.125	0.004	−0.670	−0.177
Family health → Health literacy → Frailty	−0.332	26.25	0.142	0.034	−0.597	−0.032
Family health → Physical activity → Frailty	−0.095	7.50	0.047	0.005	−0.232	−0.033
Direct effect (Family health → Frailty)	−0.837	66.17	0.218	0.000	−1.401	−0.549
Total effect	−1.265	100.00	0.068	0.000	−1.750	−1.005

## 4 Discussion

To our knowledge, this is the first study to investigate the effects of family health, health literacy, and physical activity on the frailty state of preoperative GC patients. The results demonstrate that family health has a direct negative effect on frailty, and health literacy and physical activity partially mediate the relationship between family health and frailty.

In this study, the prevalence of frailty among preoperative GC patients was 46.2%, with several previous studies reporting similar or lower rates (8, 30). These variations may be attributed to different national circumstances, frailty assessment tools and underlying concepts of frailty. The TFI scale utilized in this study is widely recognized for its effectiveness in the clinical multidimensional assessment of frailty, thereby enhancing the rigor and reliability of our findings. Furthermore, preoperative health promotion and prehabilitation are key strategies for improving cancer care outcomes (31) and enhance the efficiency of medical resource utilization (32). Studies have shown that early prehabilitation in preoperative frail patients is more likely to reduce surgical risks and promote long-term recovery compared to those with postoperative frailty (7). Therefore, the findings of this study provide a valuable intervention strategy for managing preoperative frailty.

The mediating effect model of this study revealed a significant direct effect between family health and frailty, with an effect size of −0.837, which accounted for 66.17% of the total effect. This indicates that a higher level of family health can effectively prevent or delay the onset and progression of frailty, acting as a protective buffer. Previous research has demonstrated a positive direct effect of family functioning on patients’ chronic disease management and health-related quality of life (33). These findings are consistent with our results, which may be attributed to factors such as emotional communication within the family, lifestyle choices of family members, access to family health resources, and external family support (24). Our study hypothesizes that positive emotional bonding among family members enhances mental resilience and coping skills in patients with GC, thereby improving their frailty condition. Chew et al. (34) also found that healthy family emotional dynamics foster resilience and are linked to improved mental and physical health outcomes, such as reduced depression and chronic pain. Additionally, health habits developed in a family environment can significantly enhance patients’ health knowledge and encourage the active adoption of healthy lifestyles, ultimately improving overall health status and mitigating the progression of frailty. Furthermore, family health resources influence the availability and quality of medical support for patients facing illness (35). Strong family health resources, coupled with external social support, enable patients to access and utilize health information more effectively, thereby better managing their frailty condition. Therefore, family health plays a crucial role in the management of frailty in preoperative GC patients.

However, current frailty research predominantly concentrates on individual-level factors, such as rehabilitation exercises, nutritional optimization, multi-component interventions, and individualized geriatric care models (7, 36). This suggests that healthcare professionals should broaden their understanding of family health, optimize and strengthen the internal relationships of patients with preoperative GC, and enhance external support for patients’ families, thereby promoting effective preoperative frailty management. Moreover, as family health serves as a negative predictor of frailty risk, clinical efforts should prioritize populations with vulnerable family health, including patients from low-income backgrounds and those with unstable family structures, such as individuals living alone, single parents, and intergenerational families. Early identification of high-risk frailty groups among preoperative GC patients, along with targeted preoperative rehabilitation measures, can significantly improve frailty status.

This study demonstrated that the negative impact of family health on frailty in preoperative GC patients can be moderated by health literacy and physical activity, with a total indirect effect value of −0.428, representing 33.83% of the overall effect. This indicates that both health literacy and physical activity play a crucial role in the relationship between family health and frailty in this patient population. Previous research (37, 38) has identified health literacy as a significant mediator between family health and outcomes such as wellbeing, family burden, and self-management among chronic disease patients, aligning with the findings of this study. Rothbaum et al. (39) describe the family as a cohesive social unit that functions like a system, autonomously establishing rules and responsibilities. In this context, it is essential for families to guide members experiencing frailty in enhancing their health literacy, which is considered a fundamental family responsibility. This study found a significant negative relationship between physical activity and the risk of frailty, corroborating previous reports (27, 40). It is speculated that, given the impaired physiological reserves of preoperative patients with GC, inadequate physical activity may further elevate inflammatory biomarkers, leading to disturbances in the body’s internal environment and nutrient loss. This, in turn, can adversely affect muscle mass, function, and strength, ultimately reducing physical activity levels and body weight, thereby contributing to frailty (41).

Against the backdrop of accelerating population aging and increasingly strained healthcare resources, this study proposes that clinical staff shift their focus from difficult-to-modify frailty biomarkers emphasized in previous research to modifiable cognitive and behavioral factors. We advocate integrating family health education and family-empowered physical activity programs into preoperative frailty management protocols for GC patients. Specifically, by enhancing patients’ understanding of health knowledge and motivation for physical activity, we encourage family members to assist in delivering systematic, evidence-based physical activity programs that include resistance training, aerobic activity, and balance exercises. This intervention strategy offers both practicality and cost-effectiveness, reducing the risk of preoperative frailty while alleviating pressure on formal healthcare systems. It aligns clearly with United Nations Sustainable Development Goal (SDG) 3 “Good Health and Well-Being” (42). By positioning families as fundamental units of health promotion, our research provides scientific evidence for achieving health equity and efficient resource utilization—key objectives outlined in SDG targets 3.4 “Reduce non-communicable disease risks” and 3.8 “Achieve universal health coverage.”

## 5 Limitations and future research

This study has several limitations: first, it was conducted solely in a tertiary hospital in China, which may restrict the representativeness and generalizability of the parallel mediating effect model; future multi-center studies are recommended to improve these aspects. Second, as a cross-sectional study, while it explored the potential mechanisms by which family health influences frailty through parallel mediation analysis, the actual causal relationships require further investigation through prospective longitudinal studies. Third, the two mediating variables in this study—health literacy and physical activity—accounted for 33.83% of the total effect of family health on frailty, suggesting their roles are limited. Family health is a holistic concept associated with individual health, integrating fundamental elements such as family structure, function, and social networks (35). Likewise, frailty is a complex, multi-dimensional state influenced by various physiological, psychological, and social factors (43). Future research is recommended to broaden its scope by incorporating additional health behavior factors related to frailty, such as nutritional intake and emotional self-regulation, and to explore more potential pathways through which family health may influence frailty. This will provide a more comprehensive basis for developing family-centered, multi-collaborative preoperative frailty intervention programs.

## 6 Conclusion

The analysis of the mediating effects in this study indicates that family health has both direct and indirect predictive effects on the risk of frailty, with health literacy and physical activity serving as partial mediators. Therefore, it is recommended that healthcare professionals prioritize vulnerable groups with compromised family health, such as patients from low socioeconomic backgrounds or with unstable family structures. Implementing preoperative rehabilitation measures that focus on family involvement can guide patients in enhancing their health literacy and increasing their physical activity levels, thereby effectively reducing the risk of frailty in preoperative GC patients and promoting long-term rehabilitation outcomes.

## Data Availability

The raw data supporting the conclusions of this article will be made available by the authors without undue reservation.
